# Supplementary material for: Using human brain activity to guide machine learning
Source: Sci Rep. 2018 Mar 29;8:5397. doi: 10.1038/s41598-018-23618-6 (PMC5876362; doi:10.1038/s41598-018-23618-6)
Supplement: Supplementary file 1 — Supplementary Material [file 41598_2018_23618_MOESM1_ESM.pdf]

Supplementary Material for "Using human brain activity to guide machine learning"

Ruth C. Fong,[1,3,†] Walter J. Scheirer,[2,3†] David D. Cox[3*]

[1]Department of Engineering Science, University of Oxford
Information Engineering Building, Oxford OX1 3PJ, United Kingdom

[2]Department of Computer Science and Engineering, University of Notre Dame
Fitzpatrick Hall of Engineering, Notre Dame, IN, 46556, USA

[3]Department of Molecular and Cellular Biology, School of Engineering and Applied Sciences
and Center for Brain Science, Harvard University.
52 Oxford St., Cambridge, MA, 02138, USA

[*]To whom correspondence should be addressed; E-mail: davidcox@fas.harvard.edu.
[†]R.C. Fong and W.J. Scheirer contributed equally to this work.

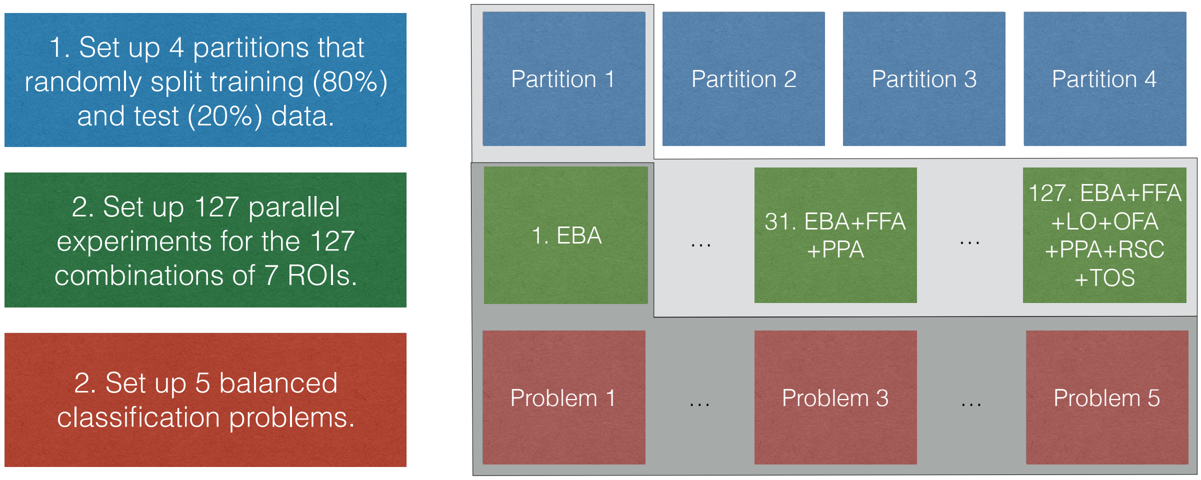

Figure 1: Experimental Design. For a given combination of ROIs (e.g., EBA+FFA+PPA), choice of image features (e.g., HOG or CNN features), and choice of loss function (e.g., hinge loss or activity weighted loss), we learn a total of 20 binary classifiers trained on image features (20 = 4 partitions $\times$ 5 balanced problems), which yield 20 classification accuracies that vary based on the sampling of partition and balanced problem data. These 20 samples are used for significance testing between HL and AWL results in Figure 2.

Supplemental Details of Experimental Set-up

Figure S1 outlines the experimental set-up. Using the set of 1,386 images that were viewed by subjects in [1], four partitions of training and test splits were randomly generated. There are 127 ways to combine the up to 7 regions of interests (ROIs) associated to high-level visual understanding (i.e., EBA, FFA, LO, OFA, PPA, RSC, and TOS) when enumerating all possible combinations of including one to seven ROIs. For each partition, each combination of ROI regions, and each of the four object categories (i.e., humans, animals, buildings, and foods), activity weights are generated for all training examples in the clear sample set by using a cross-validated classifier trained on the voxel activity from a given ROI combination (see the fMRI Activity Weight Calculation section in the main text for more details). For each partition, ROI combination, and object category, 5 balanced classification problems were set-up by randomly

sampling a partition's training set to create a balanced training set with the maximal, equal number of positive and negative examples for an object category (in practice, because we have more negative examples than positive ones, we use all positive examples and randomly sample a set of negative ones to match the number of positive examples). This balanced training set is then used to train the baseline hinge loss (HL) classifier and activity weighted loss (AWL) classifier on Histogram of Oriented Gradients (HOG) features of images in the balanced training set as well as Convolutional Neural Network (CNN) features.

Supplemental Accuracy Analysis

Additional experiments were conducted to determine what percentage of ROI combinations yielded activity weights that produced significantly better results when compared to those from baseline hinge loss classifiers. To test the significance of the difference between the classification accuracies of a hinge loss classifier and that of an activity weighted loss classifier, we conducted one-tailed, paired t-tests between two sets of 20 samples ($20 = 4$ partitions $\times$ 5 balanced problems, with the classification accuracies of a HL classifier as one set and that of an AWL classifier as the other) of the number of correctly classified instances per experiment for a given object category, ROI combination, and image feature (i.e., HOG or CNN). Not only do we observe significant improvements in classification accuracy when activity weights were generated from voxels in all 7 ROIs or from voxels in the EBA, FFA, and PPA regions, we also observe that using activity weights significantly increased classification accuracy when activity weights were generated from most of the 127 ROI combinations of voxels (Figure S2).

Supplemental ROI Analysis

The main text of the paper contains ROI influence plots (Figure 3) for biologically-informed classifiers trained with HOG features [2]. Here we present the remaining ROI influence plots for

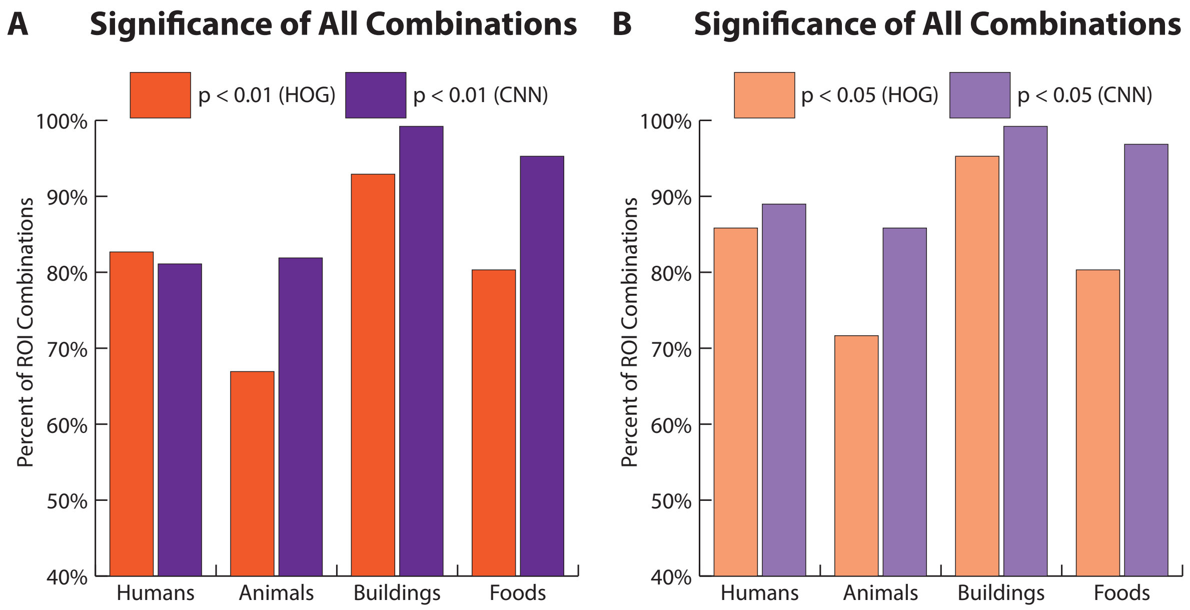

Figure 2: Significance of all combinations of ROIs. The percentage of combinations (out of 127 combinations of ROIs) in which the mean classification accuracy of classifiers that used activity weights was significantly better than that of classifiers that did not use activity weights. One-tailed, paired t-tests were used to test significance.

CNN features [3]. Figure S3 shows which ROIs significantly differed from the respective null distributions for each object category. Figures S4 and S5 demonstrate how the null distribution for this ROI significance analysis was generated. This analysis further confirms the significant impacts of the EBA region in improving the classification of humans and animals and of the PPA region in improving the classification of buildings and foods. Similar to what we observed with the HOG features, the EBA area dramatically exceeds the significance thresholds of the humans and animals null distributions.

ROI Functionality

The following list briefly describes the functional roles of the seven, higher-level ROIs used in our experiments.

1. Extrastriate body area (EBA), which is involved in recognizing the human body and body parts [4].
2. Fusiform face area (FFA), which aids in face recognition [5], although some research suggests that FFA may select for familiar items, such as cars and birds [6].
3. Lateral occipital cortex (LO), which is important for object recognition [7, 8].
4. Occipital face area (OFA), which works with FFA in face recognition by detecting individual parts of a face, such as a nose or a pair of eyes [9].
5. Parahippocampal place area (PPA), which is most active when an individual is viewing scenes or "places" [10].
6. Retrosplenial cortex (RSC), which is important for human navigation tasks and aids in episodic memory [11].

7. Transverse occipital sulcus (TOS), which is activated by visual stimuli that includes buildings [9, 12, 13].

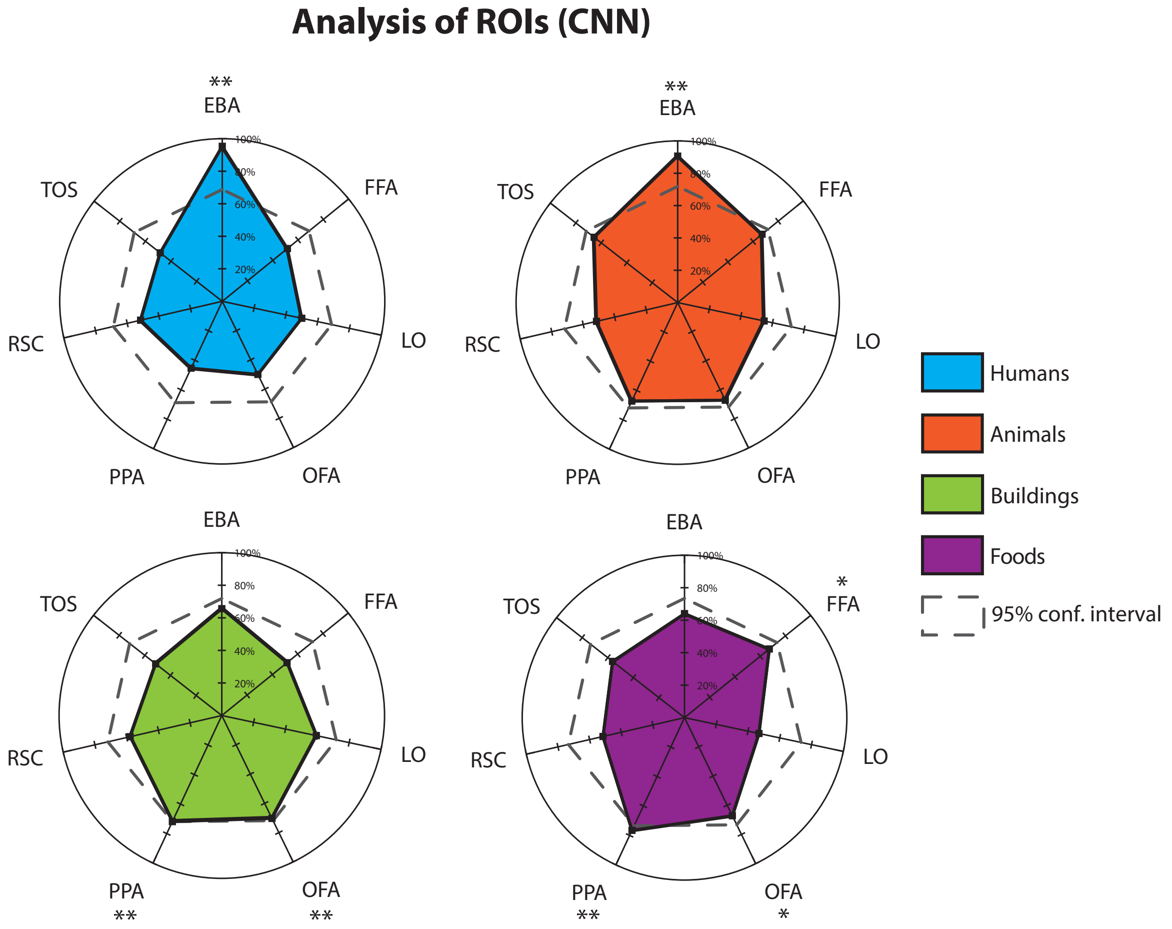

Figure 3: The influence of each ROI for the four object categories (CNN features). In each graph, the fraction of the 64 ROI combinations containing a specific ROI that had a mean classification accuracy greater than that of all 127 sets of experiments is plotted. The threshold for the 95% confidence interval ($p < 0.0004$) is also overlaid, showing which ROIs significantly differed from the respective null distribution for each object category. Bonferroni correction ($\alpha = 127$) is used to account for multiple comparisons.

		Mean Acc*	> Avg. Mean Acc (66.36%)
**1**	**EBA**	**72.02%**	**1**
**2**	**EBA, FFA**	**70.90%**	**1**
**3**	**EBA, LO**	**69.80%**	**1**
	**...**		
**64**	**EBA, FFA, LO, OFA, PPA, RSC, TOS**	**69.93%**	**1**
65	FFA	64.64%	0
66	FFA, LO	64.86%	0
67	FFA, OFA	65.67%	0
	...		
127	FFA, LO, OFA, PPA, RSC, TOS	67.15%	1
	Overall Average:	66.36%	*54.33%*
	Average for all bolded ROI combinations:	*69.91%*	98.44%

		Mean Acc*	> Avg. Mean Acc (66.36%)
**1**	**EBA**	**72.02%**	**1**
2	EBA, FFA	70.90%	1
3	EBA, LO	69.80%	1
	...		
**64**	**EBA, FFA, LO, OFA, PPA, RSC, TOS**	**69.93%**	**1**
65	FFA	64.64%	0
**66**	**FFA, LO**	**64.86%**	**0**
**67**	**FFA, OFA**	**65.67%**	**0**
	...		
127	FFA, LO, OFA, PPA, RSC, TOS	67.15%	1
	Overall Average:	66.36%	*54.33%*
	Average for all bolded ROI combinations:	*67.06%*	60.94%

Figure 4: Calculating the significance of an ROI region. **(Left)** Of the 64 ROI combinations that included the EBA region, 98.44% of them had a mean classification accuracy that was greater than the overall classification accuracy averaged over all the mean accuracies for all 127 ROI combinations (e.g., 0.6636). Note: The distribution of mean accuracies for all 127 combinations is skewed slightly left, this is reflected in the result that 69 of the 127 ROI combinations (54.33%, left table) yielded mean accuracies that were greater than the average mean accuracy over all combinations. The null distribution simply reflects this the real, skewed distribution with its mean of 0.5434 (Figure S5, left). To test the significance that the inclusion of the EBA region in the set of voxels used to learn fMRI activity weights had on improving the classification accuracy of activity-weighted classifiers, we generated a null distribution with which to compare the percentage of the 64 ROI combinations that included EBA and yielded above-average classification accuracies (e.g., 0.9844). **(Right)** A null sample for this significance permutation analysis is computed by randomly selecting 64 of the 127 regions. Then, for each of the 64 randomly selected ROI combinations, its mean accuracy is compared to the average mean accuracy over all ROI combinations (e.g., 0.6636); the percentage of the 64 ROI combinations that has a mean accuracy greater than the average mean accuracy over all ROI combinations is used as the null sample (e.g., 0.6094). Classification accuracies included in this figure are for "human" classification problem when using HOG features. '*' indicates mean classification accuracy over 20 samples (4 partitions $\times$ 5 balanced problems).

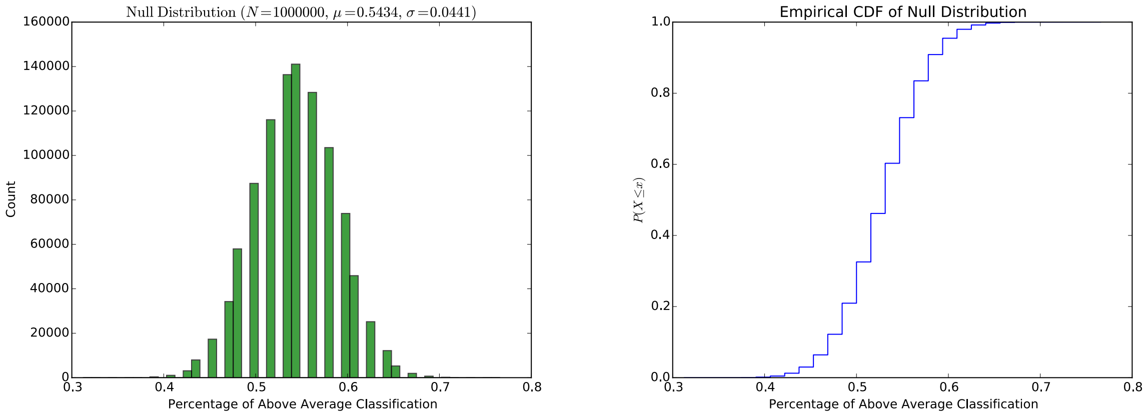

Figure 5: **(Left)** A null distribution with 1,000,000 samples generated using the procedure described in Figure S4. Then, 95% and 99% Bonferroni corrected thresholds ($\alpha = 127$) were calculated for the null distribution's empirical CDF (shown in (**Right**)), with which the real percentage of above-average classification accuracies of ROI combinations containing EBA (e.g., 0.9844 from Figure S4) were compared. Classification accuracies used in this figure are for the "human" classification problem when using HOG features, and the 95% and 99% thresholds for this setting are 0.6875 and 0.7031, which the percentage of above-average accuracies for ROI combinations including EBA well surpasses (e.g., 0.9844). (In main Figure 3, the top left plot shows the real percentage of above-average classification accuracies of ROI combinations containing each of the 7 ROIs with the 95% Bonferroni corrected confidence thresholds.)

Sequential Minimal Optimization

Sequential Minimal Optimization (SMO) [14] is commonly deployed to solve the quadratic programming problem that follows from the articulation of SVM as an optimization problem. For binary classification, assume a collection of labeled training data points $(x_1, y_1), \ldots, (x_n, y_n)$, where $x \in \mathbb{R}^d$ is a feature vector and $y \in \{-1, 1\}$ is a class label. The dual form of the quadratic programming problem for SVM is:

$$
\begin{aligned}
&\max_{\alpha} \sum_{i=1}^{n} \alpha_i - \frac{1}{2} \sum_{i=1}^{n} \sum_{j=1}^{n} y_i y_j K(x_i, x_j) \alpha_i \alpha_j, \\
&\text{subject to :} \\
&0 \leq \alpha_i \leq C, \quad \text{for } i = 1, 2, \ldots, n, \\
&\sum_{i=1}^{n} y_i \alpha_i = 0
\end{aligned}
$$

where C is a hyperparameter that controls the cost of misclassification, $K(x_i, x_j)$ is a kernel function, and α_i are Lagrange multipliers.

SMO treats the above problem as the smallest possible series of sub-problems. For any two multipliers α_1 and α_2, the constraints reduce to:

$$0 \leq \alpha_1, \alpha_2 \leq C$$

$$y_1 \alpha_1 + y_2 \alpha_2 = k$$

which can be solved analytically to find a minimum of a one-dimensional quadratic function. k is fixed on each iteration, and is the negative of the sum over the rest of the terms in the equality constraint. The SMO algorithm solves the problem via three steps: (1) find α_1 that violates the Karush-Kuhn-Tucker (KKT) conditions for the optimization problem; (2) pick α_2 and optimize the pair (α_1, α_2); (3) repeat the first two steps until the algorithm converges. To solve the entire optimization problem, this procedure must be applied until all of the Lagrange multipliers satisfy the KKT conditions. By changing the SVM loss function to Eq. 2 in the

main paper, the formulation becomes non-convex, with no guarantees on global convergence. However, by design, the optimization finds good local solutions, steered by the activity weights.

References and Notes

[1] Stansbury, D., Naselaris, T. & Gallant, J. L. Natural scene statistics account for the representation of scene categories in human visual cortex. *Neuron* **79**, 1025–1034 (2013).

[2] Dalal, N. & Triggs, B. Histograms of oriented gradients for human detection. In *IEEE CVPR* (2005).

[3] Jia, Y. & *et al.* Caffe: Convolutional architecture for fast feature embedding. *CoRR* **abs/1408.5093** (2014). URL `http://arxiv.org/abs/1408.5093`.

[4] Downing, P. E. & *et al.* A cortical area selective for visual processing of the human body. *Science* **293**, 2470–2473 (2001).

[5] Kanwisher, N., McDermott, J. & Chun, M. M. The fusiform face area: a module in human extrastriate cortex specialized for face perception. *J. Neorosci.* **17**, 4302–4311 (1997).

[6] Gauthier, I. & *et al.* Expertise for cars and birds recruits brain areas involved in face recognition. *Nature Neuroscience* **3**, 191–197 (2000).

[7] Grill-Spector, K., Kourtzi, Z. & Kanwisher, N. The lateral occipital complex and its role in object recognition. *Vision research* **41**, 1409–1422 (2001).

[8] Malach, R. *et al.* Object-related activity revealed by functional magnetic resonance imaging in human occipital cortex. *Proceedings of the National Academy of Sciences* **92**, 8135–8139 (1995).

[9] Gauthier, I. & *et al.* The fusiform face area is part of a network that processes faces at the individual level. *J. Cog. Neuro.* **12**, 495–504 (2000).

[10] Epstein, R. & Kanwisher, N. A cortical representation of the local visual environment. *Nature* **392**, 598–601 (1998).

[11] Maguire, E. The retrosplenial contribution to human navigation: a review of lesion and neuroimaging findings. *Scandinavian journal of psychology* **42**, 225–238 (2001).

[12] Hasson, U., Harel, M., Levy, I. & Malach, R. Large-scale mirror-symmetry organization of human occipito-temporal object areas. *Neuron* **37**, 1027–1041 (2003).

[13] Nakamura, K. *et al.* Functional delineation of the human occipito-temporal areas related to face and scene processing: a pet study. *Brain* **123**, 1903–1912 (2000).

[14] Platt, J. Fast training of support vector machines using sequential minimal optimization. In *Advances in Kernel Methods - Support Vector Learning* (MIT Press, 1998).
